# Layered Heterostructure of Graphene and TiO_2_ as a Highly Sensitive and Stable Photoassisted NO_2_ Sensor

**DOI:** 10.1021/acsami.4c08151

**Published:** 2024-08-07

**Authors:** Artjom Berholts, Margus Kodu, Pavel Rubin, Tauno Kahro, Harry Alles, Raivo Jaaniso

**Affiliations:** Institute of Physics, University of Tartu, W. Ostwald Street 1, Tartu 50411, Estonia

**Keywords:** CVD graphene, TiO_2_, gas sensor, NO_2_, UV light

## Abstract

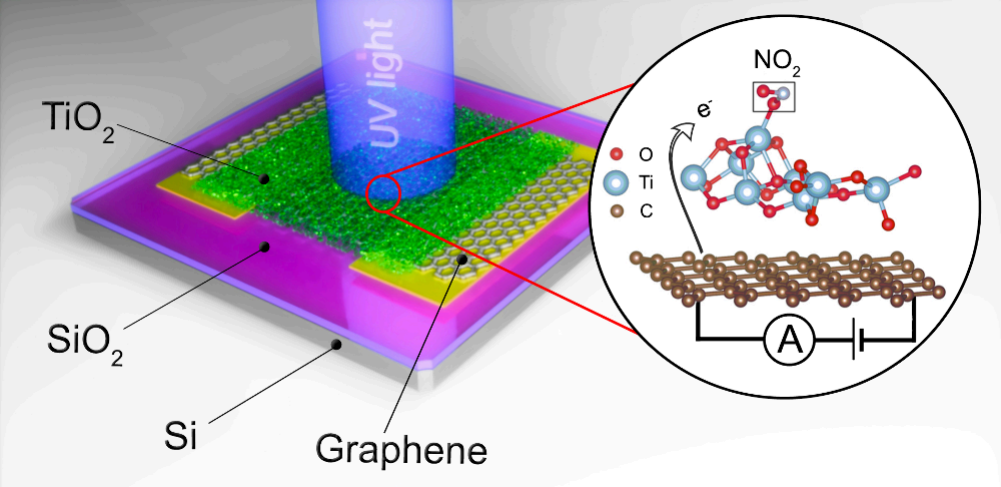

As an atomically thin electric conductor with a low density of highly mobile charge carriers, graphene is a suitable transducer for molecular adsorption. In this study, we demonstrate that the adsorption properties can be significantly enhanced with a laser-deposited TiO_2_ nanolayer on top of single-layer CVD graphene, whereas the effective charge transfer between the TiO_2_-adsorbed gas molecules and graphene is retained through the interface. The formation of such a heterostructure with optimally a monolayer thick oxide combined with ultraviolet irradiation (wavelength 365 nm, intensity <1 mW/mm^2^) dramatically enhances the gas-sensing properties. It provides an outstanding sensitivity for detecting NO_2_ in the range of a few ppb to a few hundred ppb-s in air, with response times below 30 s at room temperature. The effect of visible light (436 and 546 nm) was much weaker, indicating that the excitations due to light absorption in TiO_2_ play an essential role, while the characteristics of gas responses imply the involvement of both photoinduced adsorption and desorption. The sensing mechanism was confirmed by theoretical simulations on a NO_2_@Ti_8_O_16_C_50_ complex under periodic boundary conditions. The proposed sensor structure has significant additional merits, such as relative insensitivity to other polluting gases (CO, SO_2_, NH_3_) and air humidity, as well as long-term stability (>2 years) in ambient air. The results pave the way for an emerging class of gas sensor structures based on stacked 2D materials incorporating highly charge-sensitive transducer and selective receptor layers.

## Introduction

Nitrogen dioxide NO_2_ can be singled out as one of the principal air pollutants that is extremely harmful to humans and the environment.^1,2^ The level of this toxic gas in the air is regulated, with the EU standard limiting the NO_2_ annual mean value to 40 μg/m^3^^3^ and the US standard to 53 ppb (100 μg/m^3^ at normal conditions).^4^ The limit recommended by WHO is even stricter, being recently reduced to 10 μg/m^3^.^5^ Both national networks with stationary gas analyzers and denser networks of low-cost sensors are used to monitor NO_2_.^6−8^ Concentrations ranging from a few μg/m^3^ in the natural background up to several hundred μg/m^3^ at coal-burning industrial sources or traffic hotspots have to be measured.^6,9^ The device sensitivity must cover this concentration range, and a low susceptibility to interference from moisture and other pollutant gases and high stability are required. It has been challenging to meet all these conditions at such low NO_2_ gas concentrations with chemiresistive metal-oxide sensors; therefore, IoT-based hyperlocal monitoring mainly uses electrochemical sensors.^6−8^ At the same time, the chemiresistive sensors based on different nanomaterials are being intensively investigated and developed because of their potential for miniaturization and integration into semiconductor devices.^10−13^

Graphene is a unique two-dimensional conducting material with an ultimate area-to-volume ratio. Its electrical conductivity is highly responsive to environmental perturbations, because the density of its highly mobile charge carriers is low. Therefore, graphene is considered a very promising candidate for gas sensing applications.^11,14^ Extremely high sensitivity to different gases (down to single molecule detection) has been achieved using graphene in laboratory tests.^15,16^ In addition to the outstanding gas sensitivity of graphene and related two-dimesional (2D) materials, their relative ease of integration into CMOS devices is extremely important.^17,18^

Technological developments in semiconductor pilot lines are promising; however, in parallel, it is also necessary to open the sensing potential of 2D materials for applications. The demonstrations of the ultimate sensitivity of graphene were made in a vacuum or inert gas,^15,16^ but a critical criterion that a gas sensor material should fulfill is its ability to function in a natural air environment.^19^ In contrast to perfect transducer properties, pristine graphene is a poor, nonspecific receptor for small gas molecules. The electrical conductivity of graphene becomes sensitive to NO_2_ gas only in the presence of defects or impurities in the carbon lattice or contaminants on its surface.^15,20,21^ This is not unexpected because the binding energy of NO_2_ on the graphene surface is only 50–70 meV,^22^ while it can be several eV at the defect sites.^23^ The defects could be due to fabrication conditions,^21^ ozone treatment,^24^ grain boundaries,^25^ patterning,^26^ or impurity doping.^27^ During the past decade, several research groups have confirmed that introducing defects into the graphene lattice improves sensor performance.^14^ The decoration of graphene surfaces with nanoparticles or nanolayers of different materials has also been investigated.^28−33^

We have demonstrated that pulsed laser deposition (PLD) of silver nanoparticles or zirconia nanolayer on graphene significantly increases its sensitivity to NO_2_ gas.^30^ PLD is a universal method for deposition of various materials, such as metals, metal oxides, and other compounds, with exceptionally high precision, equal to only 1/100 of a monolayer per laser pulse.^34^ This allows us to explore an approach in which adsorption takes place on the nanolayer of another material on top of graphene but charge interchange with graphene still occurs through the interface. In this case, the sensor material is not defective graphene but a heterostructure, where graphene is an electronic transducer, and the added layer provides the receptor sites for gas molecules.

A substantial enhancement in the sensor behavior can be predicted for the TiO_2_ overlayer, because of the reduced bandgap and the variable valence states of Ti (2+,3+,4+) as compared to heavier 4d-element Zr.^35^ The bandgap values of the TiO_2_ and ZrO_2_ films are estimated to be 3.2 eV^36^ and 5.0 eV,^35^ respectively. TiO_2_ has a conduction band derived from the empty Ti^4+^ 3d orbitals, whereas the filled valence band mainly corresponds to the 2p levels of O^2–^. It is possible to optically excite TiO_2_ at ultraviolet (UV) wavelengths <390 nm, whereby the photogenerated charge carriers may invoke different surface processes (leading, e.g., to the formation of surface oxygen vacancies and Ti^3+^ ions with a lower oxidation state), and alter the chemical reactivity of the oxide surface with respect to adsorption.^36,37^ The sensitizing effect of UV light has been demonstrated in various gas-sensitive materials.^12,38^ UV-assisted sensing of NO_2_ has been investigated with sputtered TiO_2_ thin films, and a dramatic improvement in the response to 500 ppm of NO_2_ has been observed.^39^ We have previously tested this approach with other transition metal oxides (V_2_O_5_ and CuMn_2_O_4_), preferentially rendering graphene sensitive to the reducing gases NH_3_ and H_2_S.^40,41^ With TiO_2_ placed on graphene, the photoinduced effects could be more prominent and more easily achievable owing to the well-known photosensitizer properties of this oxide.^36,37^ From a practical point of view, on the one hand, the light source increases the complexity and cost of the sensor. On the other hand, without additional energy provided by a microheater or light source, the room temperature sensors^10^ have frequently very slow signal recovery and may not function stably enough in outdoor conditions.

In this study, we investigated the influence of PLD-fabricated TiO_2_ nanolayers on the electronic and gas sensing properties of chemical vapor deposited (CVD) graphene, the sensing mechanisms in the graphene-TiO_2_ (Gr/TiO_2_) heterostructure, and optimized its fabrication and operating conditions for the best performance in NO_2_ environmental monitoring.

## Results and Discussion

### Characterization of Materials

The sensors were built on 10 × 10 mm^2^ SiO_2_(300 nm)/Si substrates. A sheet of CVD graphene was transferred to cover the slit between the two prefabricated gold electrodes and a TiO_2_ layer was deposited on it using pulsed laser deposition. The details of the sample fabrication, gas sensing setup, and characterization methods are described in the Materials and Methods section.

**Figure 1:**
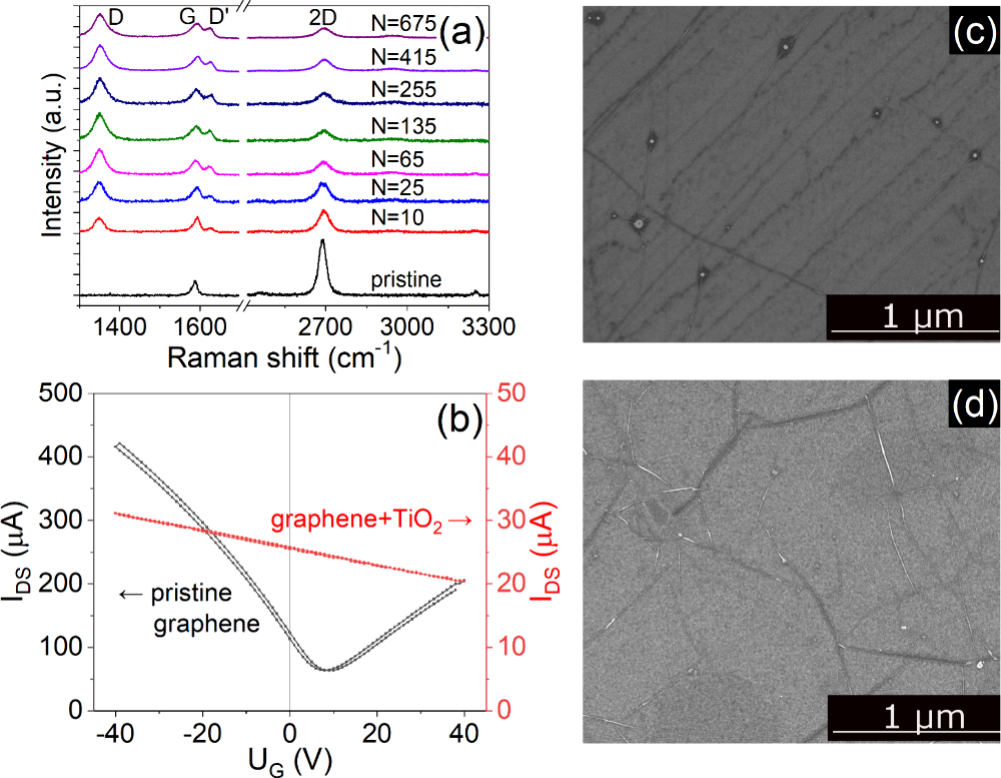
(a) Raman spectra of graphene samples with different amounts of deposited TiO_2_ (*N* denotes the number of laser pulses used in the PLD process). (b) Transconductance before and after the TiO_2_ deposition. SEM images of (c) pristine graphene and (d) TiO_2_-coated graphene.

Figure 1(a) shows the Raman spectra of pristine and TiO_2_-coated graphene (Gr/TiO_2_) samples obtained after the deposition of different amounts of the target material. The sample used in this series was modified as follows: after each deposition, it was removed from the PLD chamber, characterized, and loaded again into the PLD chamber for the next deposition. The cumulative number of laser pulses *N* used for TiO_2_ deposition is shown at each spectrum. Before PLD, pristine graphene had a minimal number of defects, indicated by missing D-peak at ≈1350 cm^–1^. The ratio of G and 2D band intensities (ca. 1:3), combined with band peak positions (1590 and 2690 cm^–1^, accordingly),^42^ prove that graphene was single-layered.

After PLD, the 2D band intensity decreased, with the emergence of defect-related bands D and D′. The ratio of the D- to G-band intensities was equal to 1.25 after the first deposition and increased to approximately 1.75 at *N* = 135 or higher. All Raman bandwidths increased by ∼50% up to the TiO_2_ layer thickness obtained with *N* = 135 laser pulses and changed only slightly (5–15%) thereafter. The lower frequency part of the spectrum contained only the Raman bands of the Si substrate (see Figure S1 of the Supporting Information), the characteristic features of TiO_2_ crystalline phases were not visible, which indicates the amorphous structure of the material.

Figure 1(b) presents the transconductance curves of the graphene sensor before and after the deposition of the TiO_2_ layer. To reduce the Fermi level shift induced by oxygen and water molecules adsorbed from ambient air,^43^ measurements were conducted under dry nitrogen flow after annealing for 2 h at 150 °C. For pristine graphene, the characteristic V-shaped curve shows the Dirac point at the gate voltage *U*_*g*_ = 8 V and the field effect hole mobility of 2035 cm^2^/(V s) at *U*_*g*_ = 0 V. After deposition of ∼0.6 nm of TiO_2_, the mobility was drastically reduced to 29 cm^2^/(V s), whereas the Dirac point shifted out of the measurement scale, due to doping. For the different devices tested, the mobility was in the range of 1000–4000 cm^2^/(V s) before and 25–30 cm^2^/(V s) after the TiO_2_ deposition.

Scanning electron microscopy (SEM) images of pristine graphene and Gr/TiO_2_ (sample with *N* = 675) are displayed in Figures 1(c) and 1(d), respectively. The structure of pristine graphene is primarily defined by the topography of the Cu foil used during the growth phase,^44^ which explains the darker lines in both subpanels. The size of the crystallites in large-area polycrystalline graphene was between 1 and 10 μm (see also additional SEM images in Figure S2 of the Supporting Information). As shown in Figure 1(d), the deposited material uniformly covered the graphene, producing a granular structure on top of the graphene. The uniform coverage by C and Ti was confirmed by mapping with energy dispersive X-ray (EDX) spectroscopy (see Figure S3 of the Supporting Information).

**Figure 2:**
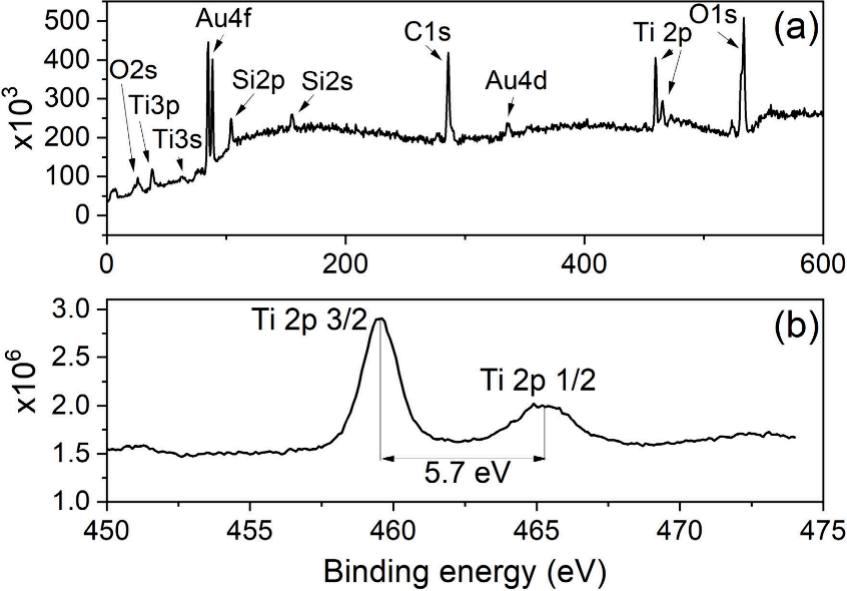
(a) XPS spectrum of TiO_2_-coated graphene on the Si/SiO_2_ substrate with Au electrodes. (b) Ti 2p spectrum.

The X-ray photoelectron spectroscopy (XPS) spectrum of a Gr/TiO_2_ sample is presented in Figure 2(a), where the lines corresponding to Ti, O, C, Si (substrate), and Au (contacts) can be observed. Generally, the XPS spectrum of TiO_2_ gives symmetric peak shapes for Ti 2p. Figure 2(b) confirms that the main form of Ti films is Ti^4+^, and the obtained spin–orbit splitting of the Ti 2p peak equals the value characteristic of TiO_2_ at 5.7 eV.^45^ Consequently, despite using the TiN target material in the PLD process, the material on top of graphene in the tested devices was TiO_2_. Presumably, oxidation occurred during exposure to air, which was facilitated by the thinness of the layer. A similar complete oxidation has been observed for a 3 nm Ti metal layer deposited on graphene.^46^

This was confirmed by the X-ray fluorescence (XRF) measurement, which resulted in mass areal densities of 0.74 mg/cm^2^ for Ti and 0.00 mg/cm^2^ for nitrogen for the *N* = 675 sample. Assuming a density of 4.0 ± 0.2 g/cm^3^ for TiO_2_ (average between rutile and anatase^36^), the thickness of the TiO_2_ layer was 3.1 nm. The ellipsometry data also agreed with the overlayer being TiO_2_ and not TiN, whereas the thickness estimate was 6.2 ± 0.4 nm at 51% porosity. These data allowed us to calibrate the growth rate at the given process parameters to 4.6 pm per laser pulse. The C 1s peak of our lab-made graphene was recently carefully analyzed, whereas the deconvolution resolved mainly sp2 carbon (87%), with some sp3 carbon (7.5%), and oxidized (C–O, C=O, O=C–O) species (5–6%).^47^

### Gas Responses

**Figure 3:**
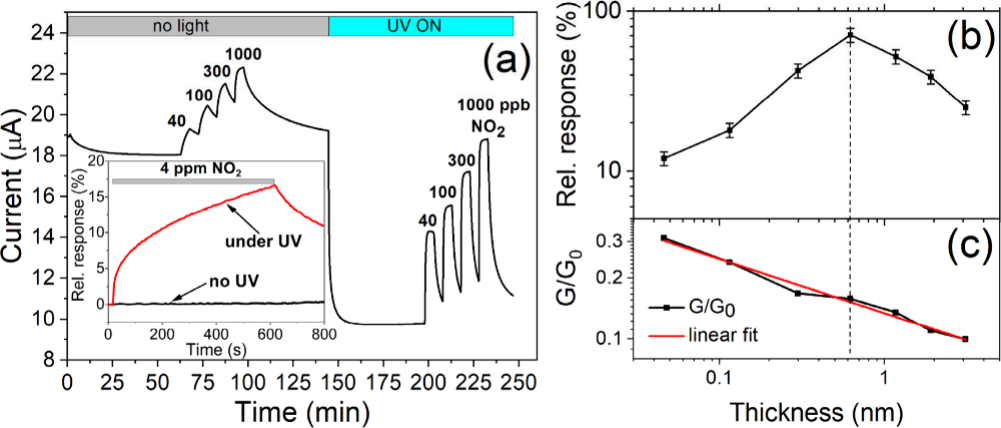
(a) Response of TiO_2_-coated graphene to different NO_2_ concentrations in the dark and under continuous UV illumination (λ = 365 nm). Measurements were performed with a supply voltage of 0.1 V at room temperature in synthetic air with RH = 20%. The thickness of TiO_2_ on single-layer CVD graphene was 0.6 nm. Inset: relative response of unmodified pristine graphene to 4 ppm of NO_2_. (b) Dependence of the relative response to 300 ppb NO_2_ and (c) the normalized conductance *G*/*G*_0_ (*G*_0_ is the conductance of the sample before PLD) on the thickness of the deposited TiO_2_ layer.

Figure 3 represents the core results of the work: it shows the drastic improvement in the gas responses of TiO_2_-coated graphene, as compared to pristine graphene, in the dark and under UV illumination. The experiments were performed in synthetic air with 20% (see Figure 3(a)) or 0% (see Figure S4 of the Supporting Information) relative humidity (RH); the UV light had an intensity of 0.2 mW/mm^2^ at 365 nm.

As shown in the inset of Figure 3(a), pristine graphene is completely unresponsive to a relatively high NO_2_ concentration (4 ppm) but acquires some sensitivity under UV illumination. An increase in the sensor’s signal (i.e., electrical current at 0.1 V supply voltage) under the influence of oxidizing gas agrees with the transconductance measurements that showed the material to be p-type, that is, hole-conducting. Graphene has been found to be p-doped by adsorbed water and oxygen molecules in air;^48,49^ our samples retained this type of conductivity to some extent even after heating in a nitrogen environment (see Figure 1(b)). Because NO_2_ acts as an electron acceptor, hole doping is promoted further, leading to increased conductivity.

The gas responses maintain their sign but are significantly amplified when the TiO_2_ overlayer coats the graphene. First, as shown in Figure 3(a), the Gr/TiO_2_ structure is already responsive in dark conditions. In this experiment, within the time interval of 62 to 102 min, four sequential exposures to NO_2_ gas at concentrations between 40 and 1000 ppb were made for 5 min each, separated by 5 min intervals of pure synthetic air. At 144 min, UV illumination was initiated, causing a significant drop in the signal. Previously, a similar effect has been observed on pristine graphene and explained by the photoinduced removal of oxygen and the associated decrease in the density of electron holes in graphene.^49,50^ Doping by oxygen has been found to depend on RH, which is ascribed to the stabilizing effect of water on oxygen anions on SiO_2_ substrates.^49^ This interpretation is consistent with the similarity of the photoinduced resistivity in pristine CVD graphene^50^ and Gr/TiO_2_ heterostructure (see Figure 3(a)).

After stabilizing the signal, a similar series of NO_2_ gas exposures were performed (see data from 200 to 250 min in Figure 3(a)). Significantly stronger and faster responses were observed with light stimulation: the relative signal change in the presence of 40 ppb test gas was approximately 10% in the dark and 40% under UV light. The repeatability of the signals under UV illumination was very good, and the responses did not differ significantly when measured in dry air (see Figure S4). The baseline returned to its former level in the dark within 2 days.

Figure 3(b) shows the effect of subsequent laser deposition (thickness of the TiO_2_ layer) on the gas response and conductivity of the Gr/TiO_2_ structure. Relative responses to NO_2_ were improved with every new PLD sequence until the titania thickness reached 0.6 nm, dropping thereafter with every new deposition. This thickness value is close to that of single-layer TiO_2_ (*d* = 0.7 nm).^51^ At the same time, a continuous decrease in conductance occurs, according to the power law *G ∼ d*^0.27^ (see Figure 3(c)). This decrease was due to the increased number of defects created in the graphene lattice during the PLD process and the interactions of graphene with the deposited material. The dependence was strongly sublinear because the already deposited material shielded graphene from the bombardment by the PLD plasma plume, and the interactions with the deposited material decreased rapidly as the distance from the graphene increased.

### DFT Simulations

**Figure 4:**
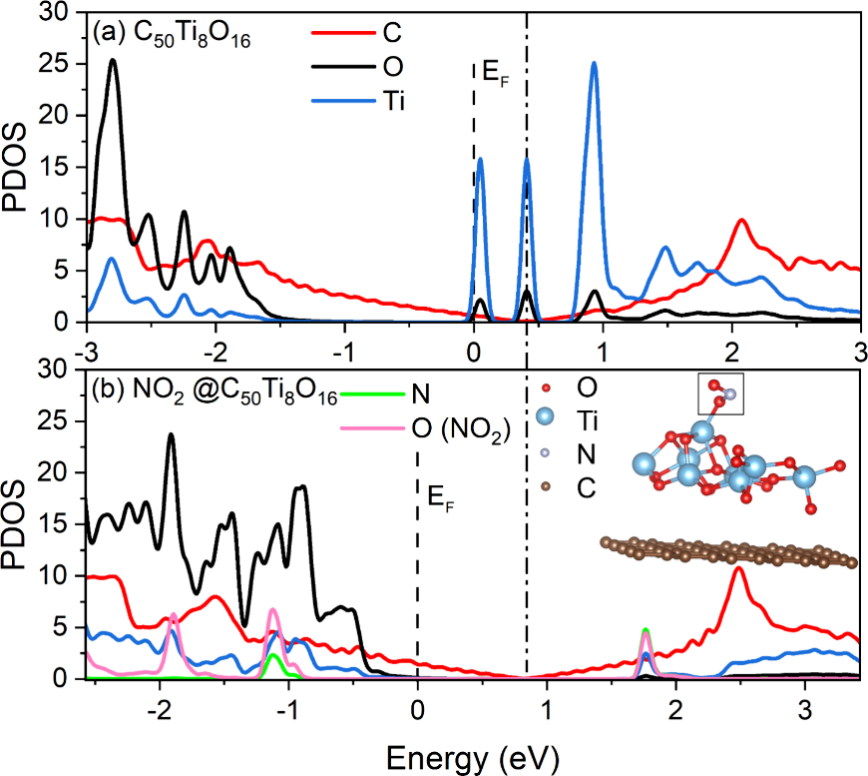
Partial densities of the states of complexes (a) Ti_8_O_16_C_50_ and (b) NO_2_@Ti_8_O_16_C_50_ were obtained using a k-mesh 25 × 25 × 1. The dashed lines indicate the position of the Fermi level. The dot-dashed line shows the position of the minima of PDOS of the carbon subsystem in the considered complexes. The inset in the lower panel shows a schematic view of the ground state geometric structure of the complex NO_2_@Ti_8_O_16_C_50_ used in DFT simulations.

To model the graphene–TiO_2_ interaction and NO_2_ gas adsorption on TiO_2_-coated graphene, we performed quasi-two-dimensional density functional theory (DFT) calculations using periodic boundary conditions. The VASP program package^52,53^ was used together with the potential projector augmented-wave (PAW) method^54,55^ and PBEsol functional approach^56^ with DFT-D3 corrections.^57^ The Bader method^58^ was employed to analyze the charge distribution in the system under consideration. First, we determined the ground states of the Ti_8_O_16_C_50_ cluster, consisting of the graphene sheet of 50 atoms and a TiO_2_ layer in a box, and an isolated NO_2_ molecule in the same box. A plane-wave basis set with a 500 eV cutoff and a Γ-point-centered mesh for k-point sampling were chosen in our calculations. For atom relaxation, k-grid sampling was taken as 4 × 4 × 1. The details of the electronic structure were investigated using a denser 25 × 25 × 1 k-point grid. All titanium, nitrogen, and oxygen atoms were fully relaxed during geometry optimization, whereas the carbon atoms were fixed in the c-direction. Next, the ground state of the complex NO_2_@Ti_8_O_16_C_50_ was determined. The final relaxed structure is shown in the inset of Figure 4 (the lattice parameters were 12.2, 12.2, and 34.3 Å). A chemical bond had formed between the oxygen belonging to the adsorbate and one of the titanium atoms. The obtained energy of the NO_2_ adsorption on Ti_8_O_16_C_50_ cluster was 2.65 eV. This value is much higher than the corresponding value of 0.055–0.067 eV in the case of pristine graphene^22^ but similar to that found for NO_2_ adsorbed on graphene defects/impurities.^23^ The Ti_8_O_16_C_50_ formation and adsorption of NO_2_ on its top led to the redistribution of the electron charge between different subsystems of the complex. Using Bader’s approach, we evaluated the changes in the Coulomb charge over the course of the reactions:
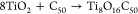
1
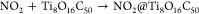
2

It was found that the electron charge transferred from the Ti and C subsystems to the adsorbate and oxygen subsystems. The values of the component charges are listed in Table 1. The charge transfer from the carbon subsystem occurred during both the formation of the complex Ti_8_O_16_C_50_ and NO_2_ adsorption. This resulted in an increase in the partial density of the electronic states (PDOS) of the carbon subsystem at the Fermi level during the adsorption process. The PDOS of all atomic subsystems of the considered complexes is shown in Figure 4.

**Table 1:** Number of Valence Electrons in the Subsystems Calculated by the Bader Method

Subsystem	Initial^a^	Ti_8_O_16_C_50_	Ti_8_O_16_C_5__0_ and NO_2_
C_50_	200.00	199.76	199.13
Ti_8_	96.00	79.38	79.22
O_16_	96.00	112.86	113.14
N	5.00		3.32
O_2_ (NO_2_)	12.00		14.19

a The number of valence electrons considered in the PAW pseudopotentials.

As can be seen in Figure 4(a), there is no Ti–C or O–C hybridization in the system under consideration: the shape of the graphene partial density of states does not change its dependency on the energy in the presence of TiO_2_ overlayer. Thus, owing to the small charge transfer between the graphene and TiO_2_ subsystems (0.24*e*, where *e* is the elementary charge; see the decrease in the C_50_ valence electron content in Ti_8_O_16_C_50_ in Table 1) and the absence of hybridization, this structure is mainly stabilized by a van der Waals interaction, which is considered in our calculations by the DFT-D3 correction.^57^ The band diagram is given in Figure S5 of the Supporting Information.

The energy scales of the two subpanels in Figure 4 are shifted with respect to each other so that the minima of the carbon subsystem PDOS (Dirac points) coincide. It is easy to see that adding a TiO_2_ cluster shifts the Fermi level toward lower energy by about 0.4 eV and the adsorption of NO_2_ by another 0.4 eV. This explains the Fermi level shift in the transconductance curves (see Figure 1(b)). The accompanying increase in the carbon PDOS at the Fermi level also explains the conductivity increase during the experimental adsorption process. The charge transferred from graphene to the adsorbed NO_2_ molecule was approximately 0.5*e* (see Table 1).

### Discussion of Molecular Mechanisms

**Figure 5:**
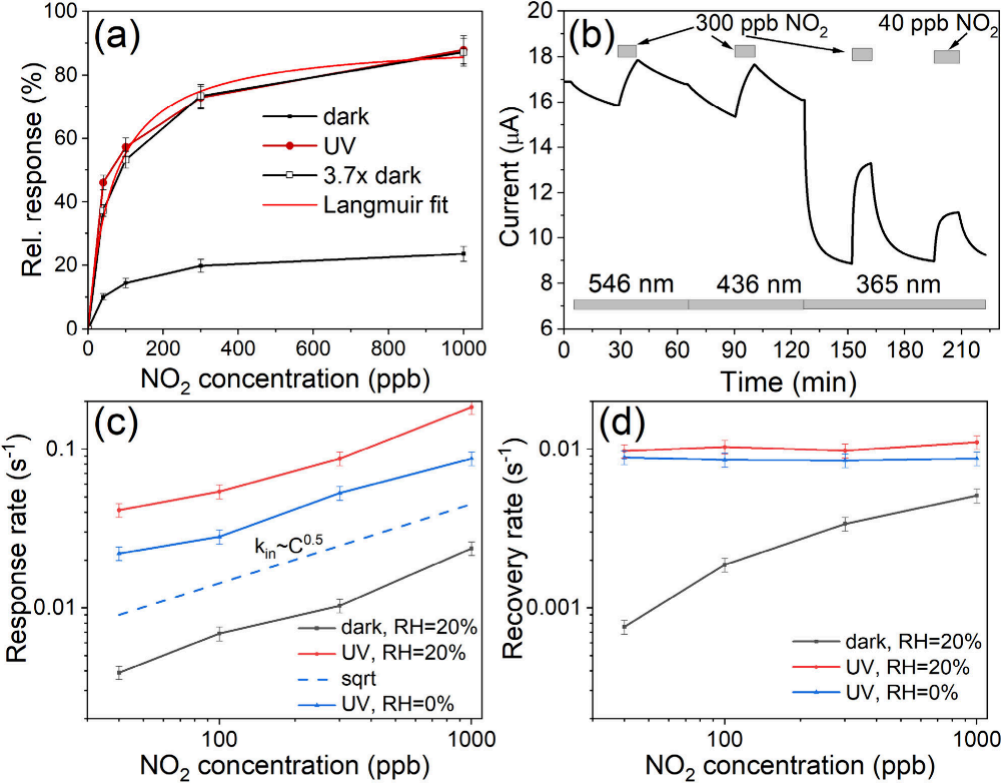
(a) Dependence of relative response on NO_2_ concentration in the dark and under UV light. The red solid line represents the approximation of UV data to the Langmuir isotherm with *c*_0_ = 65 ppb. (b) Response of Gr/TiO_2_ to NO_2_ under illumination by 546, 436, and 365 nm light. Measurement was performed in synthetic air at room temperature (RH = 20%). Dependence of (c) response and (d) recovery (desorption) rates on NO_2_ concentration as determined from the data shown in Figure 3(a) and Figure S4(a). Part of the parameters obtained from data fitting are given in Table 1 (the rates are defined as 1/*t*_*in*_ and 1/*t*_*out*_, respectively).

First, we considered the gas sensor response as a function of the NO_2_ concentration. To obtain the main parameters–response amplitudes and rates–from the data shown in Figure 3(a) and its analogs (e.g., Figure S4), each response and recovery curve was approximated with an exponential function. The dependence of the relative response amplitudes (defined as the conductance change with respect to the initial conductance) is shown in Figure 5(a). It follows that UV light improved the sensitivity by a factor of 4, and the concentration dependence resembles in both cases Langmuir isotherms,^59^ expressed as a dependence of surface coverage θ on the gas concentration *c*:
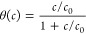
3In eq 3, the quantity *c*_0_ can be considered as the inverted adsorption–desorption equilibrium constant and, in practical terms, the concentration at which the response amplitude is half of its maximum.

To understand the key factors determining the sensitivity enhancements of graphene sensors by the TiO_2_ overlayer and UV illumination, we considered the basics of sensor signal formation. In the case of hole-dominated conductivity in graphene, the conductivity σ can be described by the following equation:
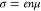
4where *n* and μ are the hole density and mobility, respectively.

Under the influence of NO_2_, conductivity is mainly affected by the charge transfer between the adsorbed NO_2_ molecules and graphene.^11^ As can be seen in Figure 3(a), the process is mainly reversible, but a fraction of the signal does not recover in the dark or has a prolonged recovery. The latter phenomenon is commonly observed in chemiresistive materials and can be explained by slow desorption from adsorption sites with high binding energies. The reversible chemisorption reaction via electron transfer is described by the following equation:
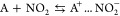
5where A is the vacant adsorption site on the surface (e.g., Ti^3+^ in titania).^60^ Consequently, the conductivity is increased by an approximate amount (neglecting the effect of mobility^61^):
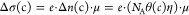
6where *N*_A_ is the density of adsorption sites and η is the fraction of electron charge extracted from graphene at the absorption of a single NO_2_ molecule.

The quantity we would like to compare (and maximize for practical applications) is the relative amplitude of the gas response, which can now be expressed by the equation:
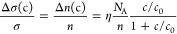
7

Let us define sensitivity *S* as the derivative of the relative response to gas concentration *c*. The sensitivity at *c*_0_ (gas concentration in the middle of the detection range) can be calculated by the following equation:
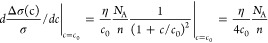
8

This quantity can be enhanced by increasing the density of adsorption centers *N*_A_ or decreasing the density of charge carriers *n* in the initial state. Generally, graphene can have high gas sensitivity owing to the low value of *n* when the Fermi level is close to the Dirac point.

However, the sensitivity is virtually absent in the case of pristine graphene (see the inset of Figure 3(a)), and in the Gr/TiO_2_ structure, the Fermi level is significantly shifted toward a lower energy below the Dirac point, which increases *n* (i.e., PDOS, contributing to conductivity). Consequently, the significantly higher sensitivity of the Gr/TiO_2_ structure can only be explained by the increased *N*_A_ due to the new adsorption centers created by the deposition of TiO_2_. The Gr/TiO_2_ heterostructure has many adsorption centers located at Ti cations, where efficient charge transfer from graphene occurs during adsorption, as confirmed by DFT simulation. Therefore, the TiO_2_ overlayer acts as a receptor, and graphene, with its conductivity generating the sensor signal, acts as a transducer.

Of course, the largest possible degree of electron transfer occurring during the elementary adsorption process is also necessary; that is, the value of η must be close to unity. The simulation using a small TiO_2_ cluster yielded a reasonably high value η = 0.5 (see Table 1). It is obvious that this factor decreases when the adsorption center is located further away from the graphene; indeed, the gas response started to decrease when the thickness of the TiO_2_ layer exceeded that of a monolayer (see Figure 3(b)).

The final parameter in eq 8, *c*_0_, is determined solely by the sensor application - its value should be in the middle of the required sensing range. For a given application, this parameter should be chosen by adequately selecting the sensor material and may be adjusted further by choosing the temperature and properties of exciting light. For environmental and air quality monitoring applications, the value of *c*_0_ corresponds to a NO_2_ gas concentration of about 50 ppb, as described in the Introduction.

The sensitivity *S* is the derivative of the curve in Figure 5(a), and it is approximately equal to 1%/ppb at the lowest gas concentrations. Another important sensor parameter, the limit of detection (LOD), can be determined from the following formula:
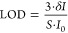
9where *δI* is the measurement noise, and *I*_0_ is the baseline signal. Because of the good electrical conductivity of graphene, the noise is relatively small; the typical ratio *δI*/*I*_0_ is equal to 10^–4^, which leads to the estimation of LOD = 0.03 ppb. A comparison of the sensitivity and LOD of different graphene-based materials is given in Table S1.

To understand the underlying mechanisms, the effects of UV light on the response and recovery curves should be considered. Under UV illumination, the response rate increased by an order of magnitude. Fitting the temporal response curves in Figure 3(a) using an exponential function yielded a response time *t*_*in*_ of 256 s in the dark vs 24 s under UV light, at 40 ppb of NO_2_. The recovery times *t*_*out*_, determined in a similar way, decreased from 1300 to 200 s, in the order of measurements from 40 ppb to 1 ppm in the dark, but were equal to 96 ± 6 s for all four curves recorded under UV light (see Table 2 and Figure 5(c,d)).

**Table 2:** Relative Response Amplitudes^a^ and Characteristic Times for Response (*t*_*in*_) and Recovery (*t*_*out*_)

	Dark			UV		
NO_2_ conc. (ppb)	(*G*_*g*_ – *G*_*air*_)/*G*_*air*_ (%)	*t*_*in*_ (s)	*t*_*out*_ (s)	(*G*_*g*_ – *G*_*air*_)/*G*_*air*_ (%)	*t*_*in*_ (s)	*t*_*out*_ (s)
40	10	256	1317	46	24	103
100	14	145	534	57	18	97
300	20	97	295	73	12	102
1000	24	42	196	88	5.4	90

a *G*_*g*_ is the conductance in the presence of NO_2_ gas, and *G*_*air*_ is the baseline conductance in air.

In the case of mutually noninteracting adsorbates on a homogeneous surface (i.e., the Langmuir model), the recovery rates (1/*t*_*out*_) should ideally depend only on the activation energy of desorption and not on the gas concentration.^59^ It follows from Table 2 and Figure 5(d) that this is not the case in dark conditions where the desorption rates increase with the gas concentration. In addition, the linearity of the adsorption rates (equal to 1/*t*_*in*_ if *t*_*in*_ ≪ *t*_*out*_) on the gas concentration, characteristic of the Langmuir process,^59^ was also not observed. Instead, a square-root dependence of 1/*t*_*in*_ on the gas concentration was observed (Figure 5(c)). A similar sublinear dependence in the case of NO_2_ adsorption on CVD graphene was ascribed to the increasing adsorption barrier with increasing coverage owing to mutual interactions between the adsorbed molecules.^62^ Such interactions also reduce the adsorbent binding energies, leading to faster desorption (higher 1/*t_out_*) at a more extensive coverage.^63^ This is exactly the behavior shown in Figure 5(d). The difference with the UV illumination where the desorption rate is higher and constant at all gas concentrations can be explained if the amount of energy released by absorbed quantum light allows molecules with even the strongest binding to cross the desorption energy barriers. The light-induced acceleration of absorption should have a different origin, as the curves under dark conditions and under UV illumination in Figure 5(c) are shifted in parallel toward higher values under illumination. This behavior is consistent with a scenario in which the light quantum does not act imminently at the adsorption, but before it liberates the adsorption sites occupied by other (competing) molecules. For example, continuous exposure of CVD graphene to UV light significantly improved the level of NO_2_ detection; a sub-ppt concentration range was achieved in an inert (ultrapure Ar or N_2_) atmosphere.^16^ The appearance of reversible oxygen sensitivity of CVD graphene under UV illumination in air, interpreted as the result of partial liberation of oxygen-binding sites, is another supporting example.^50^

We also examined the effect of visible light at wavelengths 436 and 546 nm. The respective light quanta have energies of 2.84 and 2.27 eV, smaller than the TiO_2_ bandgap (≥3 eV^36^). The behavior of the gas sensor signals under the influence of light with different wavelengths but similar photon flux is presented in Figures 5(b) and S6. The sequence of the experimental steps for the data shown in Figure 5(b) is as follows. First, the sample was kept in the dark under a constant flow of synthetic air (RH = 20%). Then, 546 nm light was applied to Gr/TiO_2_, which caused a slight decrease in the current. After an hour, the wavelength changed to 436 nm, which caused a further drop in the current at a slightly faster rate. Finally, 365 nm UV light was applied, which, in contrast to the previous two wavelengths, caused a much quicker decrease in the current and signal stabilization at about 8.75 μA. The response to 300 ppb of NO_2_ was measured under each illumination. The use of 546 and 436 nm shows smaller gas responses to 300 ppb NO_2_ compared to 365 nm (10% vs 50% signal change for 30 min). Compared with longer wavelength light, the effect of UV light on NO_2_ response and recovery times was much greater, up to 10 times.

Consequently, the onset of illumination efficiency lies in our structure between 365 and 432 nm, indicating the role of TiO_2_ as a light absorber and energy channeler. Such large differences in the influence of UV and visible light cannot be ascribed to light absorption by graphene, which covers a wide range from far-infrared to UV.^64^ We also analyzed the possibility of the direct excitation of nitrite anion NO_2_^–^, which is the most likely product formed when NO_2_ binds to the Gr/TiO_2_ surface, and found it to be negligible because of the small absorption cross-section of only about 20 M^–1^·cm^–1^.^65^ In contrast, the TiO_2_ pigment strongly absorbs UV radiation above 370 nm, whereas photoinduced charge carriers can both reduce NO_2_ and oxidize Ti^3+^ or NO_2_^–^.^36,37^ In pristine CVD graphene, the sensitizing effect of UV light has been attributed to the excitation of impurities at the Si/SiO_2_ substrate or to the removal of surface contaminants and competitors (e.g., O_2_ molecules).^66,67^

### Selectivity and Stability

**Figure 6:**
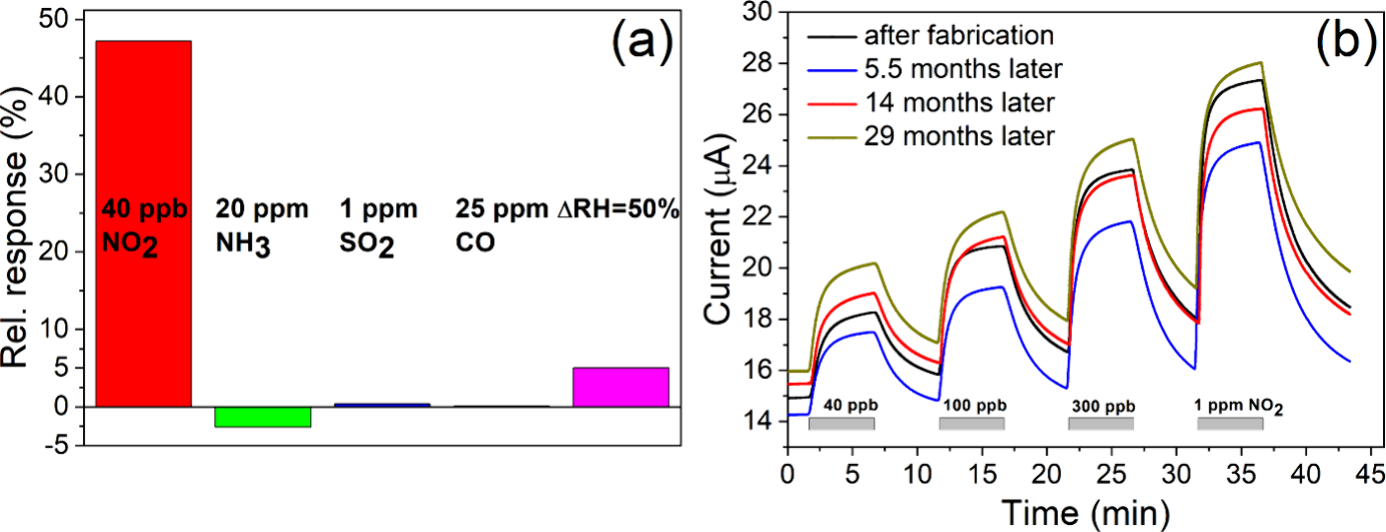
(a) Relative conductive response of Gr/TiO_2_ to different pollutant gases and humidity level changes. (b) Stability and repeatability of Gr/TiO_2_ sensor electrical conductance signal and gas responses over 29 months. Samples were stored under ambient conditions between measurements.

To determine the selectivity of the optimized Gr/TiO_2_ structures, their gas responses were measured with respect to other prominent pollutants (NH_3_, CO, and SO_2_). It follows from Figure 6(a) that the produced material can detect small NO_2_ concentrations more effectively than much higher concentrations of NH_3_ and other pollutants. These cross-sensitivity measurements were performed under UV illumination in synthetic air at a relative humidity of 20%.

The gas sensitivity can be compared with data from previous publications on Gr/TiO_2_ structures, particularly in the case of ammonia. Previous studies have reported a signal change of approximately 20% at an NH_3_ concentration of 400 ppm under visible light^46^ or at an NH_3_ concentration of 25 ppm in a biased field effect transistor.^68^ Our structures, fabricated by a different route, had a relative signal change of only a few percent for an NH_3_ concentration of 20 ppm (see the dynamic gas responses at different NH_3_ concentrations in Figure S7 of the Supporting Information). Obviously, gas sensitivity is strongly dependent on the manufacturing method.

The cross-sensitivity to the RH change was also relatively small, as shown in Figures 6(a) and S8. In Figure 6(a), the change for a 50% humidity difference is shown, whereas the supplementary Figure S8 provides a more detailed view of the signal changes compared to the NO_2_ response. Small changes in the background signal owing to humidity variations facilitate the practical use of sensors at NO_2_ concentrations below 100 ppb. A comparison of Figures 3(a) and S4 reveals that NO_2_ response amplitudes depend somewhat (up to 50%) on RH: the sensitivity at low NO_2_ concentrations is higher in humid air. The response rates exhibited a similar trend, as shown in Figure 5(c).

Long-term stability and repeatability of responses to the target gas are essential for reliable sensors. Figure 6(b) demonstrates that even without preconditioning (e.g., heating in an inert gas or vacuum), the sample exhibited excellent stability and repeatable responses to NO_2_ at concentration ranging from 40 ppb to 1 ppm over 29 months. Between the experiments, the sample used in the long term tests was held in ambient air in a laboratory room without special humidity/temperature controls.

The largest variations in the long term measurement data were observed in the initial baseline of the signal and its drift. These changes did not behave monotonically in one direction or another and were likely related to seasonal variations. The response amplitudes, as measured from the beginning to the end of each 300 s gas exposure cycle, were more stable; in the data of Figure 6(b), the variations in the amplitudes are below 10%. Another essential sensor characteristic, the limit of quantization (LOQ), can be determined based on the long-term accuracy estimate. Assuming that the baseline variability can be corrected, we estimated that the LOQ = 5 ppb.

## Conclusions

We fabricated gas sensor heterostructures based on CVD single-layer graphene and a laser-deposited TiO_2_ layer. In such a structure, the semiconducting metal oxide fulfills the function of a redox-active receptor, whereas graphene is an electronic signal transducer. By varying the deposition conditions we were able to find an optimal TiO_2_ thickness and a trade-off between the degree of graphene functionalization and the partial damage to the 2D lattice of graphene during deposition. Optimal heterostructures are well suited for detecting NO_2_ gas at concentrations below one ppm that must be monitored in the environment. The sensitivity, defined as the relative signal change per unit gas concentration, reached 1%/ppb under UV light (365 nm, 0.2 mW/mm^2^) at room temperature. Exposure to relatively weak UV light increased the sensor signal amplitude by several times and, more importantly, accelerated both the reaction and recovery kinetics by an order of magnitude. The response times were between 24 and 5 s, at 40 ppb and 1 ppm of NO_2_, respectively, whereas the recovery times were approximately 100 s at all tested gas concentrations. The effect of visible light (436 and 546 nm) turned out to be much weaker, indicating that the excitations due to light absorption in TiO_2_ play an important role, whereas the characteristics of the gas responses imply the involvement of both photoinduced adsorption and desorption. The sensor structure was highly selective to NO_2_, as compared to CO, SO_2_, and NH_3_, had a low (<5%) cross-sensitivity to humidity, and retained high sensitivity to NO_2_ for over two years. The sensing mechanism is ascribed to NO_2_ adsorption on the surface of TiO_2_, which results in electron transfer from graphene to the adsorbed gas molecules. Consequently, the Fermi level of graphene moves further away from the Dirac point, and the conductivity of the initially p-doped graphene increases. This was confirmed by DFT simulations with Ti_8_O_16_ clusters on graphene, showing both the Fermi level shift of graphene and an efficient charge transfer between the graphene and NO_2_ molecule adsorbed on the Ti cation. Experimentally, it was shown that the optimal thickness of the TiO_2_ receptor layer is about 0.6 nm, corresponding roughly to the oxide monolayer. Although it is not a crystalline monolayer but a granular coating, the result paves the way for a novel class of gas sensor structures based on 2D heterostructures with an ultimately sensitive transducer and selective receptor layer.

## Materials and Methods

### Sample Fabrication

The CVD graphene used in this study was grown on 25-μm thick polycrystalline copper foil (99.5%, Alfa Aesar) in a laboratory-built hot-wall quartz tube CVD reactor or purchased from Graphenea. To transfer it onto substrates (Si with 300 nm SiO_2_; area 10 × 10 mm^2^) with two predeposited Ti/Au (7 nm/80 nm) electrodes, graphene on Cu foil was first coated a with poly(methyl methacrylate) (PMMA; MW ∼ 996 000 g/mol, Sigma-Aldrich) layer, and then Cu was dissolved in 1 M ammonium persulfate solution. Graphene attached to the PMMA overlayer was thereafter transferred in deionized water (DI) onto the electrode substrate. Finally, PMMA was dissolved in dichloromethane (Alfa Aesar), and the sample was washed with DI and dried.

Pulsed laser deposition (PLD) was utilized to produce oxide nanolayers on top of the graphene. Prior to deposition, the sensor substrates with graphene were heated in a PLD chamber at 150 °C at a base pressure of 10^–6^ mbar for 2 h. PLD was performed at 35 °C in 0.05 mbar of N_2_ gas using a KrF excimer laser (COMPexPro 205, Coherent) with a pulse frequency of 5 Hz at an energy density of 7 J/cm^2^ for target ablation. A hot-pressed 1-in. diameter TiN sputtering target disc (Goodfellow) was used as the PLD target.

The gas pressure of 0.05 mbar in the PLD process was found to be optimal based on the maximal sensitivity of the Gr/TiO_2_ heterostructure. Graphene was significantly damaged if lower pressure was used in the PLD process. At a gas pressure of 0.01 mbar in the deposition chamber (which can be referred to as “harsh” deposition conditions), even a single laser pulse produced the D-line in the Raman spectrum with *I*_*D*_/*I*_*G*_ > 2. In refs (46 and 68) a 3–10 nm thick TiO_2_ layer was created on graphene by electron-beam evaporation (EBE; actually, Ti was deposited that later oxidized in the air), while the D-band did not appear in the Raman spectrum as a result of this process. Consequently, EBE did not produce any defects in graphene, which can be explained by the lower energy of the deposited particles than that in the PLD process used in our study. The energy of particles landing on the substrate can be reduced in PLD by increasing the gas pressure in the deposition chamber, which leads to the thermalization of the initially highly energetic plasma plume. At higher gas pressures (“soft” deposition conditions), the number of created defects decreased, as indicated by the small *I*_*D*_/*I*_*G*_ ratio and relatively small decrease in conductivity (see Figure S9). However, the relative gas response was smaller than that obtained at an optimal process pressure of 0.05 mbar. This can be explained by the fewer adsorption centers created or less effective charge transfer between graphene and adsorbed gas molecules; seemingly, certain defects created during the deposition in graphene may promote this transfer.

### Characterization Methods and Experimental Details

All gas-sensing measurements were performed using graphene-based resistors at room temperature. The volt-ampere characteristics were linear (see the example in Figure S10 of the Supporting Information). For the transconductance measurements, the back gate was provided by the Si substrate. To contact the gate, the oxide layer was removed from the back side of the Si plate using HF, which was then coated with a Ti(7 nm)/Au(80 nm) contact layer. The field-effect mobility was estimated at *U*_*G*_ = 0 using the equation μ_*FE*_ = *g* × (*L*/*W*)/(*C*_ox_ × *V*_SD_*)*, where *g* is the transconductance, *L* and *W* are the length and width of the channel, respectively, and *C*_ox_ is the capacitance of the oxide layer (300 nm thick SiO_2_) per unit area.

The gas-sensing measurement scheme is shown in Figure S11. The electrical conductance was recorded with a Sourcemeter Keithley 2400 at a fixed supply voltage of 100 mV applied to the electrodes with a gap length of *L* = 4 mm and width of *W* = 1 mm. In transconductance measurements, the second Sourcemeter provided the gate voltage between the upper Au electrode and the Si substrate. In all experiments, a constant gas flow of 200 sccm was maintained through a 7 cm^3^ sample chamber. The purity of all gases used was 99.999% (Linde Gas). All measurements were performed in synthetic air (21% O_2_ and 79% N_2_), whereas the nitrogen flow was partially directed through a water bubbler, thus allowing the regulation of relative humidity (RH) levels between 0% and 50%. The ratio between the flow rates from different gas cylinders was changed using mass flow controllers (Brooks models SLA5820) to adjust RH and NO_2_ concentrations. Gas concentrations and humidity levels were regularly calibrated by measuring the gas mixer output with an APNA-370 NO_*x*_ monitor (Horiba) and a Hygropalm 1 humidity and temperature indicator (Rotronic), respectively. A Xe–Hg lamp (L2422, Hamamatsu) with different sets of filters was used as a light source: 1) a water-filled filter to cut off the infrared part of the light; 2) a narrow-band interference filter to select the wavelength (Andover 2-in. models with central wavelengths of 365, 436, and 546 nm); and 3) grayscale filters to set the intensity of light on the sample. The same photon flux of 3.7 × 10^20^ photons/m^2^·s was maintained for all wavelengths, corresponding to 0.2 mW/mm^2^ at 365 nm. During the continuous illumination, the light power on the sample was 2.4 mW. We determined the temperature rise due to illumination to be 1.1 ± 0.3 °C at a light power of 10 mW, which allowed us to neglect the thermal effects.

Structural characterization of graphene was performed using a micro-Raman spectroscopic system Renishaw inVia at an excitation wavelength of 514 nm. The morphology of the samples was characterized using a high-resolution SEM Helios NanoLab 600 (FEI). The elemental mapping of the samples was performed using the EDX spectrometer from Oxford Instruments (Abingdon, UK), which was coupled to SEM. TiO_2_ layer thickness was deduced from XRF and ellipsometry data, recorded with a dispersive spectrometer ZXS-400 (Rigaku) and a spectrometric ellipsometer GES-5E (Semilab, Sopra), respectively. XPS measurements were conducted under ultrahigh vacuum conditions, using Mg K_a_ X-rays from a nonmonochromatic twin anode X-ray tube (Thermo XR3E2) and an electron energy analyzer SCIENTA SES-100.

## Supporting Information Available

Full range Raman spectrum of Gr/TiO_2_ on Si/SiO_2_. Wider area SEM images of lab-made and commercial graphene, EDX mapping of Gr/TiO_2_ on Si/SiO_2_, gas responses and repeatability in dry air (RH = 0), photoresponses of the baseline current in air at different light wavelengths (365, 436, and 546 nm), Gr/TiO_2_ band diagram, dynamic responses to NH_3_ and H_2_O, gas response of the sensor with TiO_2_ deposited at higher pressure, volt-ampere characteristic, scheme of gas measurement setup, and comparison table of graphene-based NO_2_ gas sensors (PDF)
