# Supplementary material for: Layered Heterostructure of Graphene and TiO2 as a Highly Sensitive and Stable Photoassisted NO2 Sensor
Source: ACS Appl Mater Interfaces. 2024 Aug 7;16(33):43827–37. doi: 10.1021/acsami.4c08151 (PMC11345727; doi:10.1021/acsami.4c08151)
Supplement: Supplementary file 1 — am4c08151_si_001.pdf [file am4c08151_si_001.pdf]

SUPPORTING INFORMATION

Layered Heterostructure of Graphene and TiO_2 as Highly Sensitive and Stable Photo-Assisted NO_2 Sensor

*Artjom Berholts, Margus Kodu, Pavel Rubin, Tauno Kahro, Harry Alles, and Raivo Jaaniso**

Institute of Physics, University of Tartu, W. Ostwald St 1, Tartu 50411, Estonia

*Email: raivo.jaaniso@ut.ee

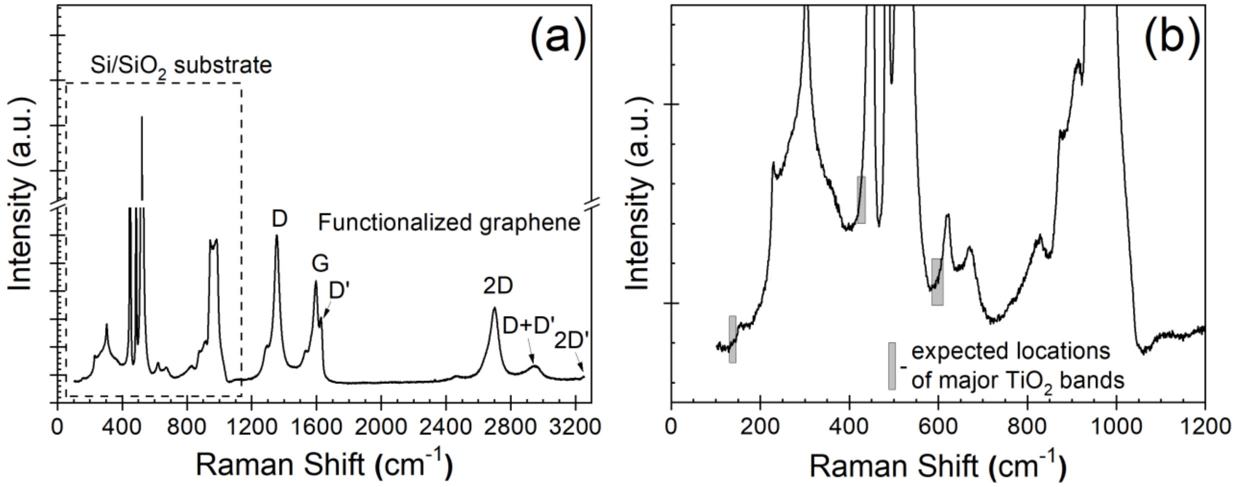

Figure S1. (a) A full range Raman spectrum of a Gr/TiO_2 sample and (b) its low frequency part showing the Si spectrum. The expected positions of the major rutile and anatase bands are marked by grey rectangles in (b).

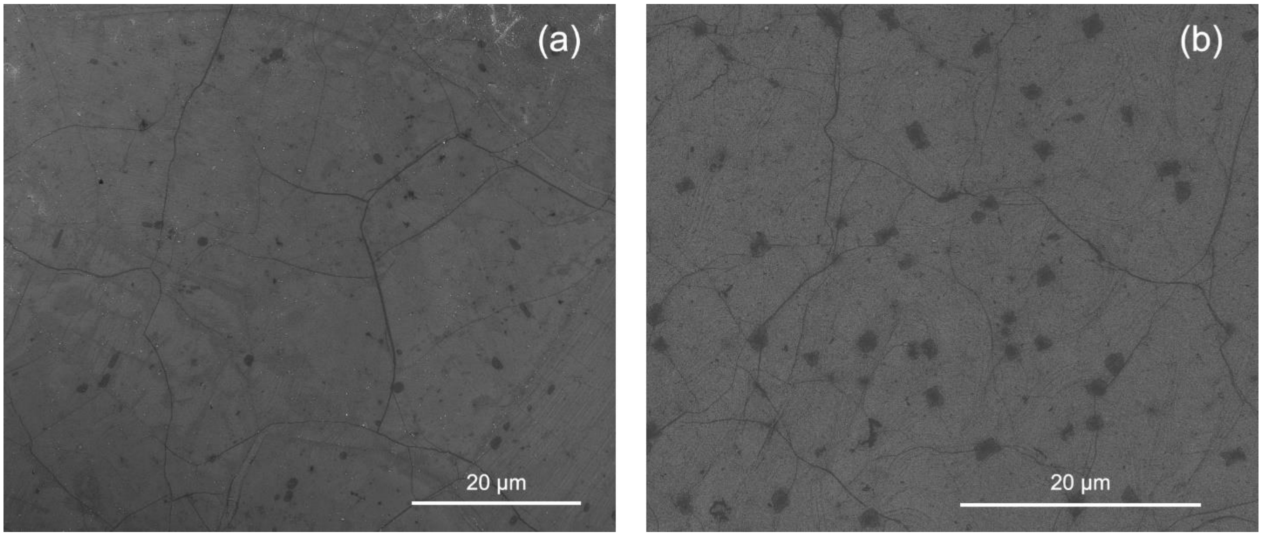

Figure S2. SEM images of (a) a lab-grown CVD graphene and (b) a commercial (Graphenea) graphene used in this study. The darker lines originate from the polycrystalline Cu foil topography or grain boundaries/wrinkles of graphene on the Si/SiO_2 substrate. The darker spots are from the multilayer graphene.

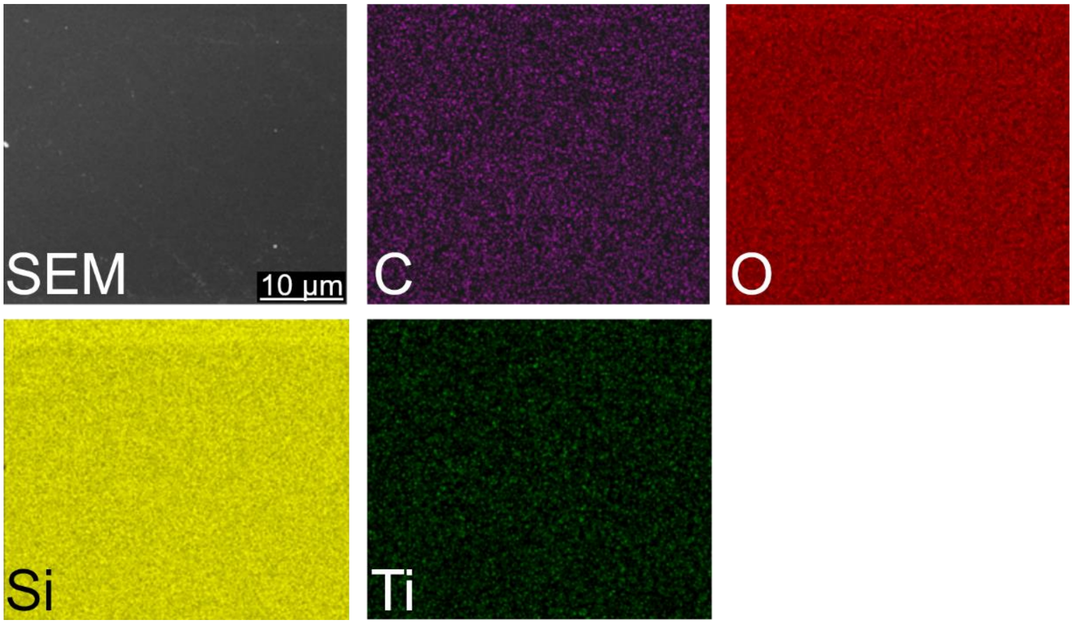

Figure S3. Elemental mapping analysis of a Gr/TiO_2 structure on the Si/SiO_2 substrate showing the electron image (SEM) and the distribution of constituent elements (C, O, Si and Ti).

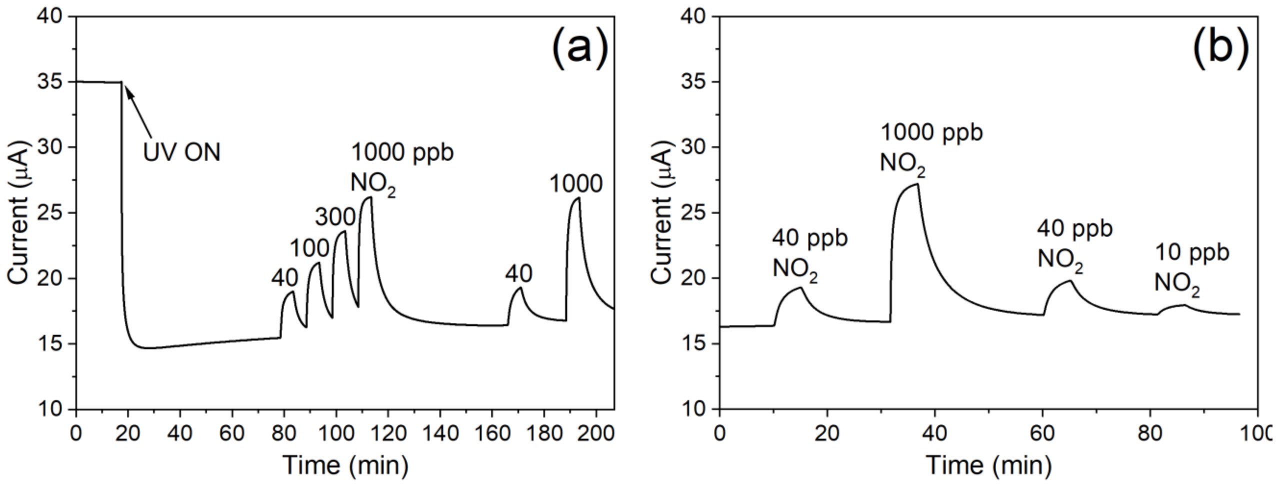

Figure S4. (a) Electrical current signal of a Gr/TiO_2 sensor with responses to different NO_2 concentrations in dry synthetic air (RH=0%) under UV illumination (365 nm, 0.2 mW/mm^2); (b) repeatability of 40 ppb response after exposure to high NO_2 concentration and a response to 10 ppb of NO_2.

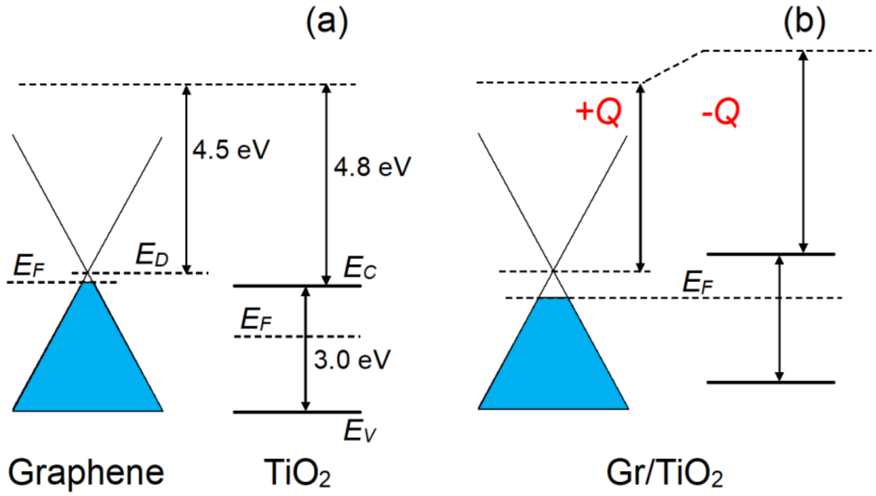

Figure S5. Band diagram of p-doped graphene and thin TiO_2 layer (a) separately and (b) as a combined system. E_F is the Fermi level, E_D is the Dirac point energy of graphene (4.5 eV below the vacuum level), and E_C and E_V are the energies of conduction band minimum and valence band maximum, respectively. The charge transferred between the subsystems in the combined system is denoted by Q.

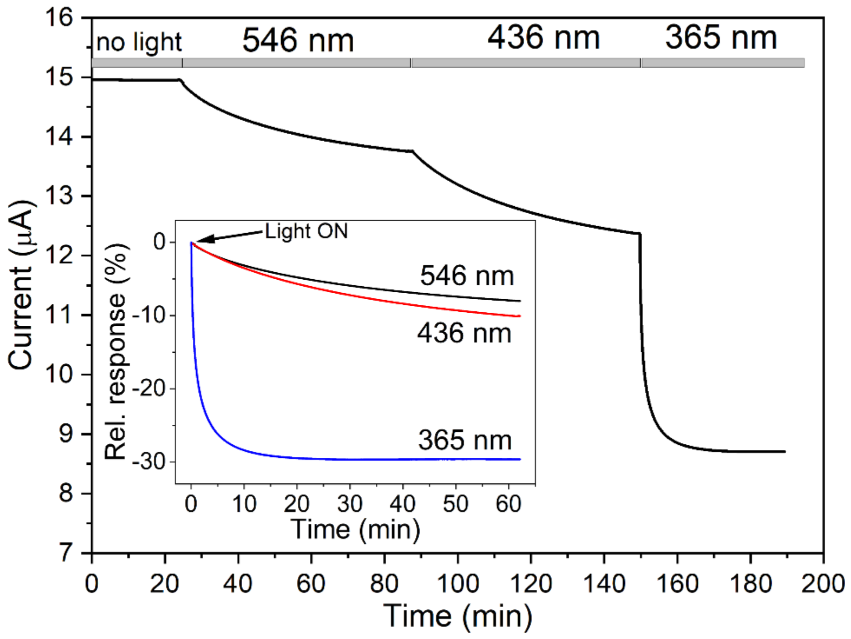

Figure S6. Photoresponses of a Gr/TiO_2 sensor under illumination at different wavelengths in synthetic air with RH=20%. The inset shows the relative change of electrical current. Illumination at the UV wavelength 365 nm improves the photoreaction rate by an order of magnitude.

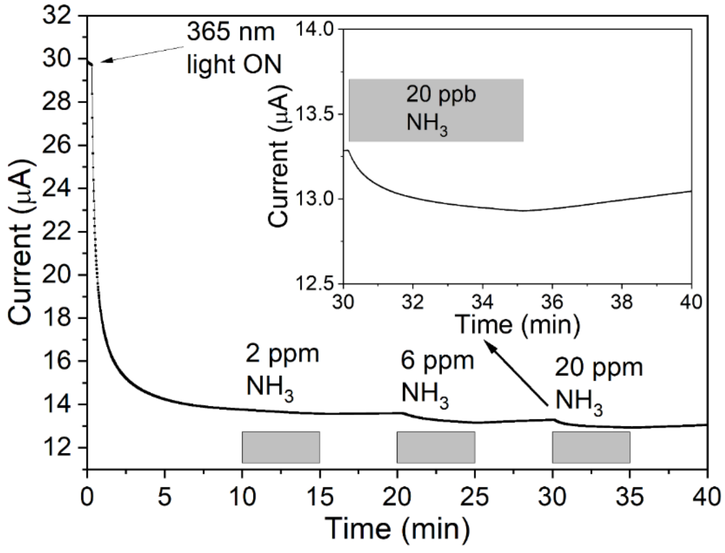

Figure S7. Response of a Gr/TiO_2 sensor to different NH_3 concentrations under illumination by 365 nm light in the synthetic air at room temperature.

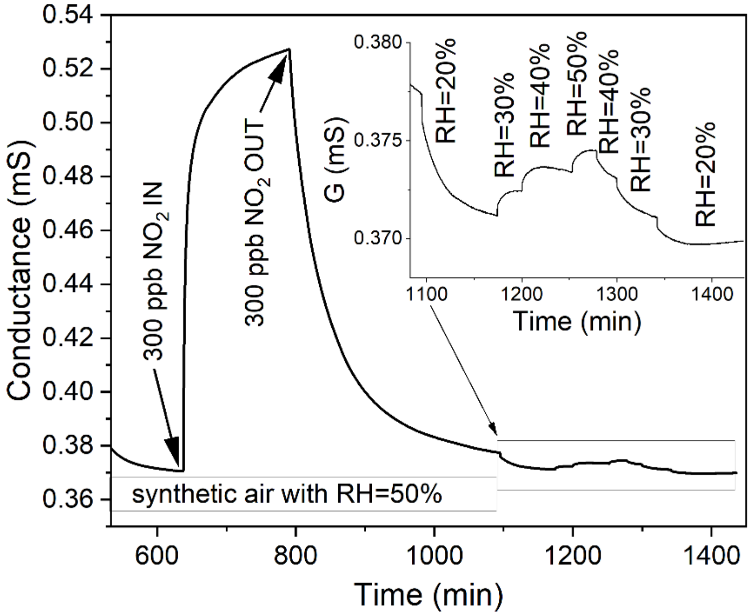

Figure S8. Effect of relative humidity (RH) on sensor signal. The cross-sensitivity to humidity is < 2% of signal change per 30% (from 20 to 50%) of RH change.

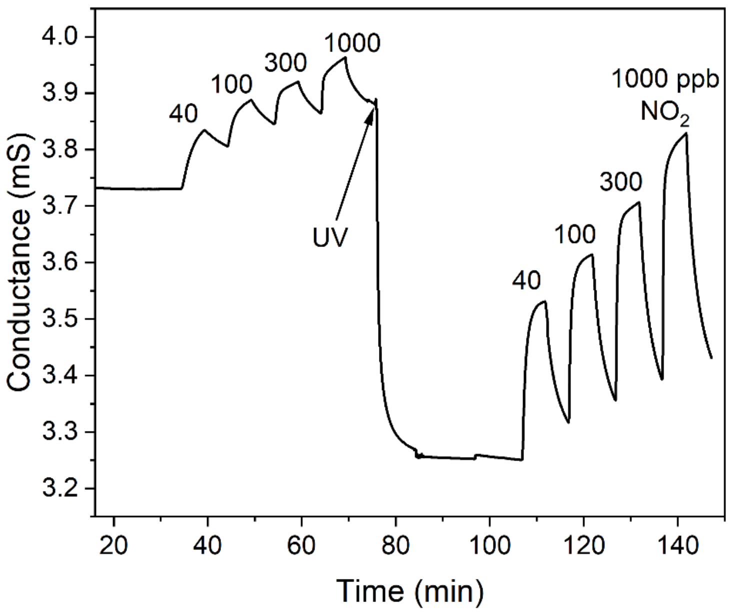

Figure S9. Conductance signal of a Gr/TiO_2 sensor with TiO_2 deposited at higher N_2 pressure (0.1 mbar) in PLD chamber. The conductance of the device before TiO_2 deposition was 9.1 mS.

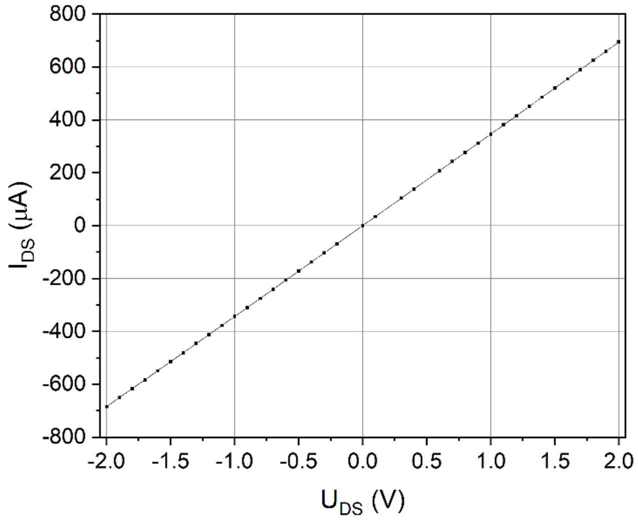

Figure S10. Volt-ampere characteristic of a Gr/TiO_2 sample.

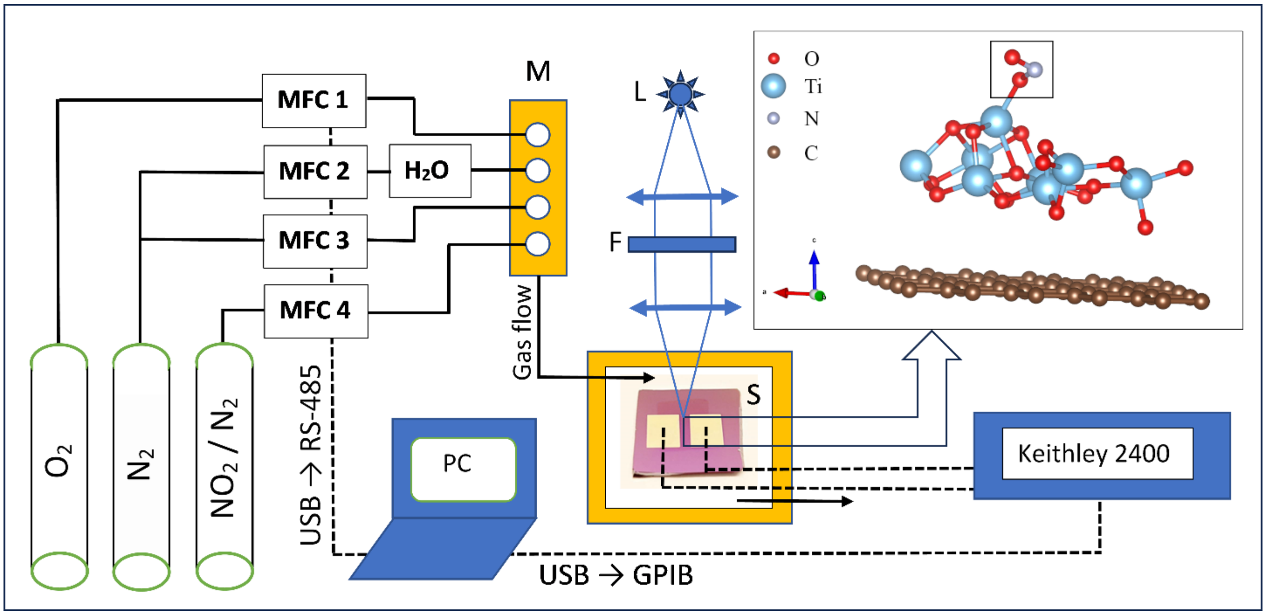

Figure S11. Measurement setup scheme with sample (S) photo and graphical atomic structure. MFC stands for mass flow controller, M is a gas mixer, L is a light source, and F is an optical filter set.

Table S1. Comparison of NO_2 sensing characteristics of graphene-based sensors tested at RT and low NO_2 concentrations*

Sensor material	Conditions (temperature, light)	Concentration range and carrier gas	Sensitivity (*S*), Limit of detection (LOD)	Response time / recovery time	Influence of humidity	Stability	Reference
**CVD graphene**							
CVDG / TiO_2 heterostructure	RT<br>UV (365 nm, 20 mW/cm^2)	10 – 1000 ppb<br>Synthetic air	*S* = 1 %/ppb (@ 40 ppb)<br>LOD = 30 ppt	24 s / 103 s (@ 40 ppb)<br>5.4 s / 90 s (@ 1 ppm)	*S* increased with RH,<br>2% baseline change @ ΔRH = 30%	Stable over 2.5 years	This work
CVDG	RT<br>UV cleaning	40 – 800 ppt<br>Ar or N_2	*S* ~ 25%/ppb (@ 40 ppt)<br>LOD = 2 ppt	≥ 10 min	-	-	[S1]
Nanopatterned CVDG	RT – 125 ºC	0.3 – 100 ppb<br>Dry synthetic air	*S* ~ 1 %/ppb (@ 1 ppb)**<br>LOD ~ tens of ppt	> 10 min / > 1 h	-	-	[S2]
Boron-doped CVDG	RT<br>UV (254 nm, 1.7 mW/cm^2)	1 – 20 ppb<br>Ar	*S* ~ 1%/ppb (@ 1 ppb)<br>LOD = 95 ppt	> 10 min	-	-	[S3]
CVDG with Pt and Ti layers	RT	20 ppb – 3 ppm<br>Dry air	*S* ~ 0.025 %/ppb (Pt; @ 20 ppb)	~ 2 min / ~10 min (@ 1 ppm)	-	S decreased 4-5 times over 20 days	[S4]
CVDG / TiO_2 Schottky junction	RT	0.1 – 3 ppm<br>Ambient air	*S* = 0.01 %/ppb (@ 100 ppb)<br>LOD = 28 ppb	~ 10 min	-	*S* decreased by 65% per month	[S5]
CVDG / ZnO	RT	100 – 1000 ppb<br>N_2	*S* = 0.06 %/ppb (@ 100 ppb)	~ 10 min	*S* decreased with RH; 5% baseline change (@ ΔRH = 70 %)	-	[S6]
CVDG with polystyrene beads	RT<br>Light (1064 nm)	0.045 – 8 ppm<br>Dry air	*S* ~ 0.04 %/ppb (@ 45 ppb)<br>LOD = 0.5 ppb	> 10 min	*S* increased with RH (~ 5 times @ ΔRH = 57 %)	*S* decreased by 25% after 4 months	[S7]

CVDG with Cu electrodes	RT<br>UV (254 nm, 0.45 mW/cm^2)	0.2 – 5 ppm<br>Air	*S* ~ 0.02 %/ppb<br>(@ 0.3 ppm)<br>LOD = 12 ppb	~ 5 min / ~ 10 min	-	-	[S8]
Few-layer CVDG	RT – 100 ºC	250 – 1000 ppb<br>Air	*S* ~ 0.025 %/ppb<br>(@ 250 ppb)<br>LOD = 5 ppb	> 1 h	*S* increased with RH	-	[S9]
CVDG / $ReSe_2$	RT<br>Light (532 nm, 310 mW/cm^2)	20 – 200 ppb<br>N_2	*S* = 2 %/ppb (@ 20 ppb)	39 s / 126 s (@ 200 ppb)	Longer recovery at RH = 20% as compared to RH = 40% and higher	*S* decreased by 20% after 1 month	[S10]
CVDG / InN nanowire barristor	RT	1 ppb – 20 ppm<br>N_2 or air	*S* = 1 - 4 %/ppb<br>LOD = 0.5 ppb	~ 1 min / ~ 4 min	Longer recovery at higher RH (range: RH = 0 – 70 %)	90% of devices stable over 1 year	[S11]
**Epitaxial graphene**							
EG	RT	1 – 50 ppb<br>Synthetic air	*S* ~ 0.1 %/ppb	> 10 min	No effect on baseline (range: RH = 0 – 20 %)	-	[S12]
EG (single and double layer)	70 ºC	10 – 154 ppb<br>Synthetic air	*S* = 1 – 2 %/ppb	~ 10 min	*S* increases with RH	-	[S13]
EG functionalized by ion implantation	RT – 150 ºC	50 ppb – 50 ppm<br>Synthetic air	*S* = 0.24 %/ppb (RT)<br>*S* = 0.72 %/ppb (150ºC)	> 10 min (RT)<br>~ 1 min / ~ 10 min (150 ºC)	-	-	[S14]
**Nanocomposites with reduced graphene oxide**							
rGO / ZnO nanorods	RT	1 – 10 ppm<br>Dry air	*S* = 0.12 %/ppb<br>(@ 1 ppm)<br>LOD = 50 ppb	75 s / 132 s (@ 1 ppm)	-	*S* decreased by 11% in 30 days	[S15]
Porous rGO	RT	20 – 1000 ppb<br>N_2	*S* = 2.44 %/ppb	10 min /<br>15 min	Baseline not affected	-	[S16]
rGO / Fe_2O_3	RT	50 ppb – 10 ppm<br>Air	*S* ~ 0.2 %/ppb	2 min / 40 min	*S* decreased with RH	-	[S17]

Sulfonated rGO / SnS_2	RT – 100 ºC	125 – 1000 ppb Air	*S* ~ 0.14 %/ppb (@ 125 ppb and RT) LOD = 0.7 ppb	~ 5 min / ~10 min (@ 125 ppb and RT)	*S* not affected in the range of RH = 30 – 90 %	Stable over 2 months	[S18]
Ag-modified rGO aerogel	RT – 235 ºC	80 – 5000 ppb Synthetic air	*S* ~ 0.1 %/ppb LOD = 6.9 ppb (@ 133 ºC)	75 s / 90 s (@ 133 ºC)	-	Stable over 30 days	[S19]
rGO / ZnO	RT	5 ppb – 10 ppm Air	*S* ~ 5 %/ppb (@ 10 ppb) LOD = 4.1 ppb	26 s / 164 s (@ 2 ppm)	*S* decreased with RH (RH = 20 – 68 %)	*S* decreased by 10% in 20 days	[S20]
EDA-rGO / SnO_2	RT	0.1 – 5 ppm Air	*S* ~ 0.05 %/ppb (@ 100 ppb)	73 s / 81 s (@ 1 ppm)	-	-	[S21]
rGO-SnO_2 / SnS_2	RT – 120 ºC	10 ppb – 10 ppm Air	*S* ~ 3 %/ppb (@ 10 ppb and 120 ºC)	42 s / 111 s (@ 10 ppm and 120 ºC)	Small effect on *S* (ΔRH = 80 %)	Stable over 8 months	[S22]
rGO / Fe_2O_3 / SnO_2	RT	0.1 – 3 ppm Dry air	*S* ~ 2.6 %/ppb (@ 100 ppb)	1 min / 34 min (@ 1 ppm)	-	-	[S23]
Phosphate-doped rGO	RT – 100 ºC	200 – 1000 ppb Air	*S* ~ 0.025 %/ppb (@ 200 ppb) LOD = 1 ppb (@ 100 ºC)	> 10 min	*S* increased with RH	-	[S24]

* Some parameters were not given directly in the references and were estimated using the data therein

** Sensitivity after three gas exposures

Abbreviations:

CVD – chemical vapor deposition

CVDG – CVD graphene

EG – epitaxial graphene on SiC

rGO – reduced graphene oxide

EDA-rGO – ethylenediamine modified rGO

RH – relative humidity

ΔRH – change in RH

RT – room temperature (usually 25 ^{o}C)

UV – ultraviolet light

References

(S1) Chen, G.; Paronyan, T. M.; Harutyunyan, A. R. Sub-Ppt Gas Detection with Pristine Graphene. *Appl. Phys. Lett.* **2012**, *101* (5), 053119. https://doi.org/10.1063/1.4742327.

(S2) Cagliani, A.; Mackenzie, D. M. A.; Tschammer, L. K.; Pizzocchero, F.; Almdal, K.; Bøggild, P. Large-Area Nanopatterned Graphene for Ultrasensitive Gas Sensing. *Nano Res.* **2014**, *7* (5), 743–754. https://doi.org/10.1007/s12274-014-0435-x.

(S3) Lv, R.; Chen, G.; Li, Q.; McCreary, A.; Botello-Méndez, A.; Morozov, S. V.; Liang, L.; Declerck, X.; Perea-López, N.; Cullen, D. A.; Feng, S.; Elías, A. L.; Cruz-Silva, R.; Fujisawa, K.; Endo, M.; Kang, F.; Charlier, J.-C.; Meunier, V.; Pan, M.; Harutyunyan, A. R.; Novoselov, K. S.; Terrones, M.

Ultrasensitive Gas Detection of Large-Area Boron-Doped Graphene. *Proc. Natl. Acad. Sci. U.S.A.* **2015**, *112* (47), 14527–14532. https://doi.org/10.1073/pnas.1505993112.

(S4) Zhao, M.; Dong, F.; Yan, L.; Xu, L.; Zhang, X.; Chen, P.; Song, Z.; Chu, W. High Efficiency Room Temperature Detection of NO_2 Gas Based on Ultrathin Metal/Graphene Devices. *RSC Adv.* **2016**, *6* (87), 84082–84089. https://doi.org/10.1039/C6RA16863A.

(S5) Falak, A.; Tian, Y.; Yan, L.; Zhao, M.; Zhang, X.; Dong, F.; Chen, P.; Wang, H.; Chu, W. Room Temperature Detection of NO_2 at Ppb Level and Full Recovery by Effective Modulation of the Barrier Height for Titanium Oxide/Graphene Schottky Heterojunctions. *Adv. Mater. Interfaces* **2019**, *6* (22), 1900992. https://doi.org/10.1002/admi.201900992.

(S6) Alfano, B.; Miglietta, M. L.; Polichetti, T.; Massera, E.; Bruno, A.; Di Francia, G.; Delli Veneri, P. Improvement of NO_2 Detection: Graphene Decorated With ZnO Nanoparticles. *IEEE Sensors J.* **2019**, *19* (19), 8751–8757. https://doi.org/10.1109/JSEN.2019.2922412.

(S7) Fei, H.; Wu, G.; Cheng, W.-Y.; Yan, W.; Xu, H.; Zhang, D.; Zhao, Y.; Lv, Y.; Chen, Y.; Zhang, L.; Ó Coileáin, C.; Heng, C.; Chang, C.-R.; Wu, H.-C. Enhanced NO_2 Sensing at Room Temperature with Graphene via Monodisperse Polystyrene Bead Decoration. *ACS Omega* **2019**, *4* (2), 3812–3819. https://doi.org/10.1021/acsomega.8b03540.

(S8) Zhao, M.; Falak, A.; Tian, Y.; Yan, L.; Liu, R.; Chen, W.; Wang, H.; Wu, T.; Chen, P.; Chu, W. Cu/Graphene Interdigitated Electrodes with Various Copper Thicknesses for UV-Illumination-Enhanced Gas Sensors at Room Temperature. *Phys. Chem. Chem. Phys.* **2020**, *22* (44), 25769–25779. https://doi.org/10.1039/D0CP04405A.

(S9) Deokar, G.; Casanova-Cháfer, J.; Rajput, N. S.; Aubry, C.; Llobet, E.; Jouiad, M.; Costa, P. M. F. J. Wafer-Scale Few-Layer Graphene Growth on Cu/Ni Films for Gas Sensing Applications. *Sens. Actuators B: Chem.* **2020**, *305*, 127458. https://doi.org/10.1016/j.snb.2019.127458.

(S10) Nazir, G.; Rehman, A.; Hussain, S.; Hakami, O.; Heo, K.; Amin, M. A.; Ikram, M.; Patil, S. A.; Din, M. A. U. Bias-Modified Schottky Barrier Height-Dependent Graphene/$ReSe_2$ van Der Waals Heterostructures for Excellent Photodetector and NO_2 Gas Sensing Applications. *Nanomaterials* **2022**, *12* (21), 3713. https://doi.org/10.3390/nano12213713.

(S11) Jahangir, I.; Uddin, M. A.; Franken, A.; Singh, A. K.; Koley, G. Investigation of Graphene/InN Nanowire Based Mixed Dimensional Barristors with Widely Tunable Schottky Barrier for Highly Sensitive Multimodal Gas Sensing Applications. *Sens. Actuators B: Chem.* **2023**, *379*, 133238. https://doi.org/10.1016/j.snb.2022.133238.

(S12) Novikov, S.; Lebedeva, N.; Satrapinski, A.; Walden, J.; Davydov, V.; Lebedev, A. Graphene Based Sensor for Environmental Monitoring of NO_2. *Sens. Actuators B: Chem.* **2016**, *236*, 1054–1060. https://doi.org/10.1016/j.snb.2016.05.114.

(S13) Melios, C.; Panchal, V.; Edmonds, K.; Lartsev, A.; Yakimova, R.; Kazakova, O. Detection of Ultralow Concentration NO_2 in Complex Environment Using Epitaxial Graphene Sensors. *ACS Sens.* **2018**, *3* (9), 1666–1674. https://doi.org/10.1021/acssensors.8b00364.

(S14) Kaushik, P. D.; Rodner, M.; Lakshmi, G. B. V. S.; Ivanov, I. G.; Greczynski, G.; Palisaitis, J.; Eriksson, J.; Solanki, P.; Aziz, A.; Siddiqui, A. M.; Yakimova, R.; Syväjärvi, M.; Yazdi, G. R. Surface Functionalization of Epitaxial Graphene Using Ion Implantation for Sensing and Optical Applications. *Carbon* **2020**, *157*, 169–184. https://doi.org/10.1016/j.carbon.2019.09.071.

(S15) Xia, Y.; Wang, J.; Xu, J.-L.; Li, X.; Xie, D.; Xiang, L.; Komarneni, S. Confined Formation of Ultrathin ZnO Nanorods/Reduced Graphene Oxide Mesoporous Nanocomposites for High-Performance Room-Temperature NO_2 Sensors. *ACS Appl. Mater. Interfaces* **2016**, *8* (51), 35454–35463. https://doi.org/10.1021/acsami.6b12501.

(S16) Peng, H.; Li, F.; Hua, Z.; Yang, K.; Yin, F.; Yuan, W. Highly Sensitive and Selective Room-Temperature Nitrogen Dioxide Sensors Based on Porous Graphene. *Sens. Actuators B: Chem.* **2018**, *275*, 78–85. https://doi.org/10.1016/j.snb.2018.08.036.

(S17) Zhang, B.; Liu, G.; Cheng, M.; Gao, Y.; Zhao, L.; Li, S.; Liu, F.; Yan, X.; Zhang, T.; Sun, P.; Lu, G. The Preparation of Reduced Graphene Oxide-Encapsulated a-Fe_2O_3 Hybrid and Its Outstanding NO_2 Gas Sensing Properties at Room Temperature. *Sens. Actuators B: Chem.* **2018**, *261*, 252–263. https://doi.org/10.1016/j.snb.2018.01.143.

(S18) Huang, Y.; Jiao, W.; Chu, Z.; Wang, S.; Chen, L.; Nie, X.; Wang, R.; He, X. High Sensitivity, Humidity-Independent, Flexible NO_2 and NH_3 Gas Sensors Based on SnS_2 Hybrid Functional Graphene Ink. *ACS Appl. Mater. Interfaces* **2020**, *12* (1), 997–1004. https://doi.org/10.1021/acsami.9b14952.

(S19) Li, Q.; Chen, D.; Miao, J.; Lin, S.; Yu, Z.; Han, Y.; Yang, Z.; Zhi, X.; Cui, D.; An, Z. Ag-Modified 3D Reduced Graphene Oxide Aerogel-Based Sensor with an Embedded Microheater for a Fast Response and High-Sensitive Detection of NO_2. *ACS Appl. Mater. Interfaces* **2020**, *12* (22), 25243–25252. https://doi.org/10.1021/acsami.9b22098.

(S20) Chen, Z.; Guo, H.; Zhang, F.; Li, X.; Yu, J.; Chen, X. Porous ZnO/rGO Nanosheet-Based NO_2 Gas Sensor with High Sensitivity and Ppb-Level Detection Limit at Room Temperature. *Adv. Mater. Interfaces* **2021**, *8* (24), 2101511. https://doi.org/10.1002/admi.202101511.

(S21) Zheng, S.; Sun, J.; Hao, J.; Sun, Q.; Wan, P.; Li, Y.; Zhou, X.; Yuan, Y.; Zhang, X.; Wang, Y. Engineering SnO_2 Nanorods/Ethylenediamine-Modified Graphene Heterojunctions with Selective Adsorption and Electronic Structure Modulation for Ultrasensitive Room-Temperature NO_2 Detection. *Nanotechnology* **2021**, *32* (15), 155505. https://doi.org/10.1088/1361-6528/abd657.

(S22) Zheng, S.; Li, Y.; Hao, J.; Fang, H.; Yuan, Y.; Tsai, H.-S.; Sun, Q.; Wan, P.; Zhang, X.; Wang, Y. Hierarchical Assembly of Graphene-Bridged SnO_2-rGO/SnS_2 Heterostructure with Interfacial Charge Transfer Highway for High-Performance NO_2 Detection. *Appl. Surf. Sci.* **2021**, *568*, 150926. https://doi.org/10.1016/j.apsusc.2021.150926.

(S23) Zhang, Y.; Yang, Z.; Zhao, L.; Fei, T.; Liu, S.; Zhang, T. Boosting Room-Temperature Ppb-Level NO_2 Sensing over Reduced Graphene Oxide by Co-Decoration of α-Fe_2O_3 and SnO_2 Nanocrystals. *J. Colloid Interface Sci.* **2022**, *612*, 689–700. https://doi.org/10.1016/j.jcis.2022.01.009.

(S24) Hasanov, B. E.; Casanova-Chafer, J.; Deokar, G.; Gouveia, J. D.; Nematulloev, S.; Gomes, J. R. B.; Llobet, E.; Costa, P. M. F. J. Amplified Sensing of Nitrogen Dioxide with a Phosphate-Doped Reduced Graphene Oxide Powder. *Carbon* **2024**, *226*, 119207. https://doi.org/10.1016/j.carbon.2024.119207.
